# Evolving roles of circadian rhythms in liver homeostasis and pathology

**DOI:** 10.18632/oncotarget.7065

**Published:** 2016-01-28

**Authors:** Dexi Zhou, Yaqin Wang, Lu Chen, Leijuan Jia, Jie Yuan, Mei Sun, Wen Zhang, Peipei Wang, Jian Zuo, Zhenyu Xu, Jiajie Luan

**Affiliations:** ^1^ Laboratory of Clinical Pharmacy of Wannan Medical College, Wuhu, Anhui Province, China; ^2^ Department of Pharmacy in Yijishan Hospital of Wannan Medical College, Wuhu, Anhui Province, China

**Keywords:** circadian rhythms, liver metabolism, epigenetic modifications, liver fibrosis, hepatocellular carcinoma

## Abstract

Circadian clock in mammals is determined by a core oscillator in the suprachiasmatic nucleus (SCN) of the hypothalamus and synchronized peripheral clocks in other tissues. The coherent timing systems could sustain robust output of circadian rhythms in response to the entrainment controlled environmentally. Disparate approaches have discovered that clock genes and clock-controlled genes (CCGs) exist in nearly all mammalian cell types and are essential for establishing the mechanisms and complexity of internal time-keeping systems. Accumulating evidence demonstrates that the control of homeostasis and pathology in the liver involves intricate loops of transcriptional and post-translational regulation of clock genes expression. This review will focus on the recent advances with great importance concerning clock rhythms linking liver homeostasis and diseases. We particularly highlight what is currently known of the evolving insights into the mechanisms underlying circadian clock. Eventually, findings during recent years in the field might prompt new circadian-related chronotherapeutic strategies for the diagnosis and treatment of liver diseases by coupling these processes

## INTRODUCTION

Circadian rhythms are generated by an endogenous molecular pacemaker [1], arising from an adaptation of 24-hour light-dark (LD) cycle derived from the earth's rotation [2]. They are capable of generating oscillatory behavior independent of external factors, for example light intensity/temperature-entrainable cues [3-5]. Further evidence supports that circadian clock programs multiple behavioral and physiological processes, such as metabolism, growth, development and other functions of diverse organisms ls [6, 7]. that Nevertheless, the disturbance of circadian rhythms appears to trigger the loss of homeostasis throughout the body because of the altered oscillation of different clock genes and CCGs [8, 9].

At the cellular and molecular levels, the circadian clock consists of many oscillators that form the feedback timing circuit. Specifically, autoregulatory negative feedback loops have been observed in mammals [10], which is commonly controlled by the crosstalk between positive and negative clock components [11]. These oscillators programme the 24-hour rhythms which are crucial for maintaining various cells and tissues-specific functions [12]. In fact, the molecular clock in all organisms shares some defined characteristics and mechanisms, including transcription/translation, post-transcriptional and post-translational modifications in regulating the feedback cycles [13, 14]. Notably, local metabolic rhythms represent an output of tissue-based circadian clocks, together with various cells characteristics [15]. Clinical and experimental animals studies have uncovered that considerable s genes, proteins and enzymes levels in livers are controlled by circadian rhythms to a great extent [16-18]. Tong et al. previously performed a systematic review to estimate the contribution of circadian rhythms to the liver physiology and diseases [19]. This would explain the findings, such as a reprogramming of the liver metabolism and transcription pathways observed in the experimental subjects with loss of circadian rhythms [20].

This review attempts to summarize the recent observations with emphasis on the potential mechanisms underlying the regulation of liver-specific circadian clock. We will clarify how circadian clocks have incorporated the clock-generating machinery into the robust output of liver circadian rhythmicity from physiology to pathology. Hopefully, this could shed light on the development of some novel therapeutic and diagnostic targets of liver diseases on the basis of circadian-related chronotherapeutic strategies.

## THE COMMON STRUCTURE OF THE CIRCADIAN PACEMAKER

The timing of daily rhythms in anticipation of the light-dark cycle is based mainly on the circadian clock synchronizes behavioral and physiological processes. It is clearly suggested that circadian rhythms can be modified by external events, includingLD cycle, temperature changes or availability of food [21-23]. Simulated daily body temperature rhythms of mice and humans are the importantly external cues in resetting and synchronizing the peripheral oscillators [24]. The impacts of temperature changes on the plant circadian clock have been observed in Arabidopsis thaliana, which could influence the clock genes expression [25]. Accumulating studies have found that some complex networks in part mediating the interplay between different clock genes by using several pharmacological or genetic interventions [26]. Growing evidence suggests an interlocked transcription-translation feedback loop as the molecular mechanism to drive circadian clock [27]. This feedback circuit is composed of a series of core clock genes, which are divided into positive elements/promoters including circadian locomotor output cycles kaput (CLOCK), brain and muscle Arnt-like protein 1 (BMAL1), and negative elements/repressors including three period (PER1, 2 and 3) and two cryptochromes (CRY1 and 2) molecules [28]. CLOCK and BMAL1 are two subunits of the heterodimeric basic-helix-loop-helix-PAS (PER-ARNT-SIM domain)-containing transcription factors (TFs). Firstly, the CLOCK and BMAL1 proteins dimerize, bind to the E-boxes (CACGTG) nucleotide sequences in the promoter of downstream targets, including the clock genes and CCGs, and thereby activates the transcription of PER and CRY. Then, PER and CRY are translated in the cytoplasm and form the PER-CRY complexes, which eventually translocate from the cytosol to the nucleus and inhibit CLOCK-BMAL1-dependent transcription activity. Importantly, the phosphorylation and ubiquitination of the negative elements leads to the low levels and degradation of PER and CRY [29, 30], in turn, allowing the positive elements at a high level to restart the new cycle. Thus, the imbalance of the production and degradation of PER and CRY either lengthens or shortens the circadian period of the clock in mammals. On the other hand, there is an alternative loop comprised by accessory proteins or nuclear receptors (NRs), including the retinoic acid-related orphan receptors (RORα, β and γ) and REV-ERB orphan receptor family (REV-ERBα and β) [31, 32]. RORα and REV-ERBα compete for the ROR response element (RORE) binding site in the BMAL1 promoter, REV-ERBα firstly accumulates quickly and inhibits BMAL1 transcription, then RORA, which accumulates more slowly and activates BMAL1 transcription, reinforce stability and robust rhythmicity of the internal clock systems [33]. Moreover, the mechanisms employed for mammalian circadian control are increasingly observed by additional post-transcriptional regulation and epigenetic modifications, apart from the classic transcription-translation pattern, which will be elucidated as following section.

**Figure 1:**
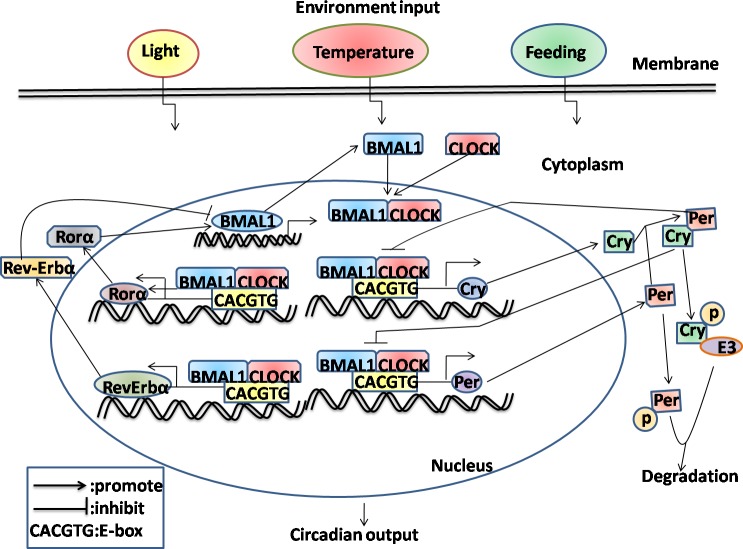
Schematic representation of molecular interactions in mammalian circadian transcriptional negative feedback loops For simplicity, many cells and tissues have the capacity to oscillate with a wide variety of periodicities, and the circadian oscillators can be entrained to local time in response to environmental stimuli including daylight, temperature and feeding availability. Firstly, the interactions between positive elements (BMAL1 and CLOCK) and negative elements (CRY and PER) which form the interconnect negative feedback loop in mammals through inhibiting CLOCK-BMAL1-dependent transcription. Then, RORα and Rev-ERBα, which form the secondary loop that regulates rhythms, resulting from the activation and inhibition the expression of BMAL1, respectively. Finally, phosphorylation and ubiquitination of the negative components results in their eventual degradation, allowing the positive components to restart the cycle.

In summary, these data suggest that proper functioning of the circadian system determined by the orchestration of a central clock in the SCN and synchronized peripheral clocks in local tissues, with emphasis on multiple regulatory mechanisms in response to the environmental changes (Figure 1).

## MOLECULAR MECHANISMS CONTROLLING CIRCADIAN RHYTHMS

The molecular clocks in mammals are existed not only in the central pacemaker neurons of SCN, but also in almost all peripheral tissues. Analysis of central and peripheral clock mechanisms has received considerable evidence that the circadian behavior is controlled at both transcriptional and post-transcriptional levels, including cellular pathways [34], TFs [35], epigenetic changes [36] and post-transcriptional regulators [37], as reviewed recently [38]. Such regulation alone or in combination has been shown to predominantly alter the phase and amplitude of rhythmic mRNA and protein expression in the liver microenvironment [39].

In the past few years, it has been rapidly uncovered that cellular signaling pathways responsible for coupling physiological and metabolic cues to the molecular clock [40]. The mTOR signaling pathway is strictly linked with the metabolic processes and nutrient avialibility in livers [41]. By the intermittently intraperitoneal injections(i.p.) of rapamycin(inhibitor of mTOR), the weight gain is largely prevented in mice on high-fat diet (HFD) than other schedules [42]. According to the Khapre et al.’ results, mTOR complex 1(mTORC1) activity oscillation was controlled by the feeding-entrainable clock mechanism confirmed by the time-resticeted (TR) feeding schedule [43]. Moreover, they further indicated that increased activity of mTORC1 signaling owing to the BMAL1 deficiency and BMAL1-dependent mTOR up-regulated mechanisms were associated with the accelerated aging [44]. In addition, many studies have also suggest connetions between cellular signal transduction and circadian cycles. For example, the insulin-phosphatidylinositol 3-kinase (PI3K)-and Forkhead box class O3 (FOXO3)-dependent signaling are required for protecting circadian rhythmicity in the liver and modulating hepatic metabolic function *via* transcriptional regulation of CLOCK [45]. Furthermore, a series of core clock and output genes (BMAL1, ARNTL and PER1,2) have been identified with the anti-tumor effects which are subject to the regulation through a non-genetic route, e.g. epigenetic changes [46].

Epigenetic modifications, including DNA methylation, non-coding RNAs and histone modifications, have been implicated to hamper the transcription and post-transcription of target genes expression, including circadian genes. Recent advances in genomic technologies have allowed researches of determining methylation of CpG dinucleotides in the promoter sequence of circadian genes. Notably, the variability of individual timing system of daily circadian behavior is influenced by environmental changes, such as the prolonged or shortened light-dark cycle, which is driven by global alterations in promoter DNA methylation in the SCN [47]. Paired specimens from the cancerous and noncancerous tissues indicate the possible disruption of the promoter DNA methylation of circadian clock genes in the development of tumorigenesis [48]. Particularly, accumulating evidences have shown that miRNAs function as the direct and indirect modulators which are the significant players in regulating various aspects of circadian clock function [49]. Finally, a variety of histone modifications patterns, for example, histone lysine demethylase JARIDa, deacetylase SIRT1, have a nonredundant role in keeping circadian oscillator function [50, 51]. The relationship between epigenetic genetics and circadian rhythms may promote the understanding of mammalian health and diseases.

## CIRCADIAN RHYTHMS IN LIVER HOMEOSTASIS AND METABOLISM

Liver is a primary target involved in the regulation of several key metabolic parameters including the levels of glucose, lipid, bile acid and other aspects of physiology [52, 53]. Circadian clocks are endogenous oscillators, driving the rhythmic expression of a broad array of clock genes and CCGs, and circadian misalignment can evoke the disparate pathologies of liver [9, 54]. Emerging evidence indicates a more integral mechanism for the coordination of circadian rhythm in orchestrating liver metabolism and physiology [55, 56].

Glucose and lipid metabolism as the major output of the circadian clock in mice liver, are associated with a dynamic protein-DNA interactome by targeting BMAL1 [57]. Adiponectin, a well-recognized antidiabetic adipokine, is involved in glucose and lipid metabolism, which more recently has been reported to be activated by BMAL1 and CLOCK through the transcriptional activity of peroxisome proliferator-activated receptor γ (PPAR-γ) and its co-activator 1α (PGC-1α) [58]. It protects from impeded insulin signaling due to some crucial signaling molecules including insulin receptor substrates (IRS) in the liver [59]. Adiponectin metabolic pathway components and expression of clock genes in liver exhibit circadian rhythmicity under low-fat diet [60], however, fasting and high-fat diet lead to phase advance and delay, respectively. Consequently, high-fat diet correlates with the malfunction of circadian rhythm, which may lead to the development of hepatic insulin resistance and obesity [61, 62]. In turn, insulin is a major regulator of FOXO activity, which is the TFs of CLOCK, indicating the insulin-FOXO3-CLOCK signaling pathway is critical for the modulation of circadian rhythms [45]. Likewise, the insulin-mTORC2-AKT signaling promotes the *de novo* lipogenesis through regulating the hepatic metabolic function of BMAL1 [63]. Hence, we could infer that hepatic circadian clock systems are highly responsive to internal cues, such as insulin metabolism [64]. The impact of circadian rhythms on hepatic metabolism has been implicated in mice with genetic deletion of different clock genes. Male PER1/2/3 triple-deficient mice gain significantly more body mass than wild-type controls on high-fat diet [65]. Similarly, knockout of the two CRY genes (CRY1, 2) in mice causes the altered dimorphic liver metabolism, along with disruption of sex-specific liver products and growth hormone (GH) [66]. Lipidomic analysis reveals circadian oscillations of hepatic triglyceride (TAG) levels, but its phases and levels are completely different in clock-disrupted or nighttime restricted feeding mice [67]. Given the deposition of excess TAG within hepatocytes as a hallmark of nonalcoholic fatty liver disease (NAFLD), indicating the NAFLD is obviously associated with a loss of circadian rhythm. Currently, many studies demonstrate circadian clock controls hepatic metabolism mostly at the transcriptional level by synchronizing the expression of liver enzymes [68]. For example, mice deficient in Nocturnin, a gene that encodes a circadian deadenylase, have deficits in lipid metabolism or uptake and changes in glucose and insulin sensitivity [69]. Remarkably, Nocturnin has been shown to enhance PPAR-γ activity [70], which is recommended at the center of a regulatory loop between circadian networks and metabolic output. It is also interesting to note that leucine-rich repeat-containing G protein-coupled receptor 4 is a new regulator for energy metabolism and mutant Lgr4^m/m^ mice show altered rhythms of TAG and cholesterol [68]. In addition, mice lacking a secondary 12 hr period rhythm characterized by rhythmic activation of the IRE1α pathway in the endoplasmic reticulum (ER), which leads to the deregulation of ER-resident enzymes and perturbed lipid metabolism through the aberrant sterol responsive element binding protein (SREBP) [71]. Conversely, fibroblast growth factor 21 (FGF-21), a hormone exists in liver and fat, dose dependently reduces body weight and hepatic steatosis is largely associated with inhibition of nuclear SREBP-1 [72]. Gain-and loss-of-function studies in mice hepatocytes showed that hepatic FGF-21 levels are suppressed by the expression of PGC-1α, which is dependent on REV-ERBα and the ligand ALAS-1 expression [73]. Similar to PGC-1α, PGC-1β knockout mice develop abnormal circadian activity, hepatic steatosis and increased serum TAG and cholesterol levels induced by high-fat feeding [74]. These results highlight the role of PGC-1α/β play in directing hepatic energy metabolism *via* the circadian-controlled manner. The CLOCK gene influences the circadian rhythms of hepatic glycogen synthesis through transcriptional activation of glycogen synthase 2 (Gys2) [75]. Eventually, sleep disruption significantly abolishes liver clock rhythms and dramatically alters hepatic bile acid (BA) metabolism by suppressing Cyp7A1 expression [76], which is correlated with the inhibited activity of TFs including hepatocyte nuclear factor 4α (HNF4α) and D-site binding protein (Dbp). However, the krüppel-like factor (KLF)15and FGF15 signaling axis promotes and inhibits the circadian BA production, respectively, which is identified as a non-hepatic basis for regulation [77].

Recent evidence indicates that epigenetic regulation appears to be participate in the circadian regulation of liver metabolism [78]. The histone H3-lysine-4 methyltransferase mixed-lineage leukemia 3 (MLL3) and its closest homolog, MLL4 complexes, named MLL3/4 complexes, function as major epigenetic regulators and pivotal coactivators of the circadian TFs, ROR-α and -γ, in the hepatic circadian control of BA production [79]. The NAD^+^-dependent protein deacetylase, sirtuin 1(SIRT1), is involved in regulating hepatic insulin sensitivity *via* CLOCK/BMAL1-dependent manner [80]. SIRT1 prevents obesity-induced hepatic steatosis by regulating lipid homeostasis through positively binding PPAR-α and coactivator PGC-1α [81]. Therefore, we might have a hypothesis that SIRT1-mediated PPAR-α/PGC-1α signaling pathway links the regulation of receptors like REV-ERBα [73]. As expected, the action and oscillation of NRs in liver may contribute to circadian entrainment of nutrient and energy metabolism [74, 82]. REV-ERBα and β are highly coordinated for protecting both clock and metabolic functions [83], dual depletion of them by creating double-knockout mice profoundly disrupts circadian expression of core circadian clock and lipid homeostatic gene networks [84]. The liver-specific microRNA, miR-122, has been found to be the major circadian transcriptional target of REV-ERBα [85]. As a result, miR-122 has emerged as key contributor of liver homeostasis and energy metabolism by the circadian-dependent manner, such as miR-122 inhibits circadian Nocturnin expression [86]. Genetic disruption of the NRs corepressor (NCOR) 1- histone deacetylase (HDAC) 3 interaction in mice causes aberrant regulation of clock genes and results in abnormal circadian and metabolic behaviour [87]. Furthermore, genomic recruitment of HDAC3 by REV-ERBα displays a circadian rhythm gene expression that is required for normal lipid homeostasis in mice liver [88].

Herein, although the advances in understanding of the circadian clocks controlling the rhythmic expression of a large number of genes involves in hepatic metabolism and homeostasis, further investigation remains to be necessary for clarifying the mechanisms of clock-controlled output systems in liver (Table 1).

**Table 1:** Circadian rhythms control hepatic metabolism and physiology

Substance	Function	Regulatory genes	Mechanisms	References
Adiponectin	Antidiabetic adipokine	*Bmal1,Clock, Ppar-γ, Pgc-1α*	Transcriptional	[58-60]
Triglyceride	Energy storage	*Nocturnin, Lgr4, Ppar-, Fgf-21, Pgc-1α and β, Srebp-1, Sirt1, miR-122*, HDAC3	Transcriptional, Epigenetics	[63, 67-74, 85, 87, 88]
Glucose	Energy supply	*Bmal1,Clock, Gys2*	Transcriptional	[75]
Insulin	Antidiabetic hormone	*Bmal1,Clock,Sirt1*	Transcriptional, Epigenetics	[80]
Bile acids	Facilitate digestion and absorption	*Cyp7A1,HNF4α, Dbp, MLL3/4*	Transcriptional, Epigenetics	[76, 77, 79]

## CIRCADIAN RHYTHMS IN LIVER INJURY AND FIBROSIS

The liver is frequently exposed to diverse insults, such as chemicals, alcohol, viral infection and metabolic disorders [89-91]. Although it could regenerate after acute injury, chronic liver damage causes fibrosis and cirrhosis. Liver fibrosis is a common scarring response to all forms of chronic liver injury [92], which can result in the increased extracellular matrix (ECMs) protein production by the activated hepatic stellate cells (HSCs) [93]. There is increasing evidence to support that the disruption of circadian rhythm is a critical molecular mechanism in the pathogenesis from organic injury to fibrosis [94-96]. Previous researches indicated that the liver expressed a diverse set of genes in a circadian manner [97] and the different genes functions were under direct or indirect circadian control [98]. Importantly, the regulatory structure that governs circadian dynamics within the liver at a transcriptional level has been uncovered by using bioinformatics analysis [97].

Elevated results in the induction of multiple liver injury responses support circadian rhythms are correlated with the magnitude of hepatic damage. Chemotherapeutic agent cyclophosphamide is known to have the anti-cancer toxicity in liver, whereas selenium compounds show protective roles agnist it by modulation of the molecular time-keeping system through the up-regualtion of BMAL1 expression [99]. Chronic alcohol exposure damages core properties of the circadian clock system which confers vulnerability to alcohol-induced pathology in liver [100, 101]. Utilizing genetically or environmentally circadian disruption models, such as CLOCK mutant and shifted LD cycle mice with chronic alcohol consumption, numerous reports demonstrate that circadian disruption promotes alcohol-induced intestinal hyperpermeability, endotoxemia and steatohepatitis possibly in part by increasing tight junction protein occludin [102, 103]. Analysis of circadian gene expression in ethanol-induced liver injury shows that the extent of injury is associated with the variations of hepatic circadian genes PER1 [104]. PER1 appears to regulate expression of the endothelin axis genes including endothelin-1 (ET-1), two receptors endothelin-A (ET_A_) and endothelin-B (ET_B_) in a time-dependent manner in liver [105]. ET-1 correlates with liver function and injury, which might enhance the nitric oxide (NO) production and the blood flow in the hepatic sinusoids [106]. Lipopolysaccharide (LPS) inhibits the ET-1-induced NO production and disrupts liver microcirculation, this process could be blocked by the Rho-kinase (ROCK-2) inhibition in the liver sinusoidal endothelial cells [107]. In addition, LPS-induced liver injury is greater during the early active than the early resting period, followed by dosing-time-dependent variation in the accumulation of polymorphonuclear cells (PMN) and subsequent IL-6 production [108]. The PER2 gene is also an important component of liver circadian system and the deficiency of PER2 induces the liver injury and fibrosis during cholestasis [96]. PER2 may function in diurnal variation of acetaminophen (APAP) induced hepatotoxicity *via* modulating Cyp1A2 expression in mice [109]. However, the amplitude of PER2 is not remarkably changed in fibrotic livers, which totally infer that PER2 gene rhythmic expression could be an important contribution mechanism to liver fibrogenesis [110]. Similarly, PER2 decreases carbon tetrachloride(CCl_4_)-induced hepatotoxicity *via* the suppression of uncoupling protein-2 (Ucp*2*) expression that depends on a CLOCK-controlled PPAR-α signal transduction pathway in mice [111]. The mechanisms of CCl_4_ toxicity in rat livers are suggested to be caused through a Cyp2E1-dependent chronotoxicity [112]. In line, further study suggests that loss of PER2 predisposes liver fibrosis by increasing HSC activation and inhibiting HSC apoptosis *via* the TRAIL-R2/DR5 pathway [113]. As expected, these findings are paralleled by enhanced levels of several key fibrogenesis gene (TGF-β_1_, TNF-α, TIMP-1) expressions in PER2^−^/^−^ mice that have undergone CCl_4_ treatment and bile duct ligation (BDL) [96, 113]. Collectively, these findings provides a variety of direct or indirect evidence of the association between the PER1/2 and liver fibrogenesis. TGF-β/Smads-dependent transcriptional signaling is the important intracellular mediator in the development of liver fibrosis [114]. Notably, the Smad3 is expressed in a circadian rhythm-manner in mice liver, which depending on CLOCK/BMAL1 [115]. These findings suggests that the TGF-β/Smads signaling pathway shows the circadian expression and leads to the fibrogenesis.

Interestingly, clock genes BMAL1, CLOCK and CRY2, along with two important clock-regulated genes PPAR-α and Cyp450 lose daily rhythms and their mRNA levels are significantly decreased in the fibrotic mice induced by CCl_4_ [110]. The two important hepatic metabolic genes PPAR-α and Cyp450 [116] are significantly impaired during liver fibrogenesis [110], which imply that alterations of hepatic clock genes lead to the changes of many physiologically metabolic parameters (e.g. lipid and drug metabolism). Indeed, the circadian clock controls most metabolic pathways in liver by regulating the metabolism-related genes expression in a cyclic fashion [68, 117]. Circadian clocks represent relevant targets to consider for optimization of therapeutic schedules of cyclin-dependent kinases (CDK) inhibitors through the clock-controlled detoxification pathways [118].

**Figure 2:**
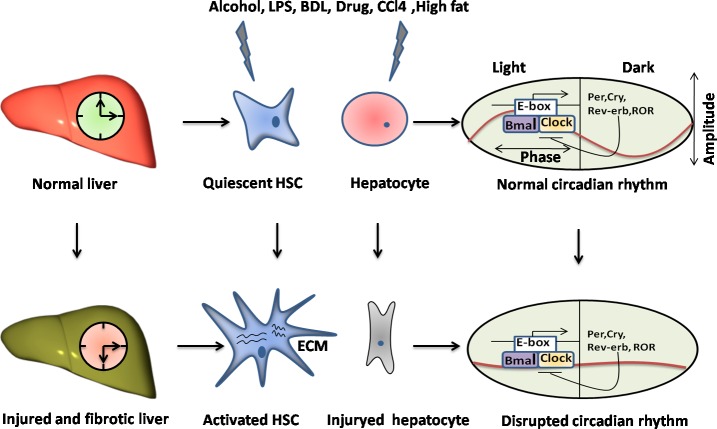
The disruption of circadian rhythms exists in the liver injury and fibrosis The livers undergo the variously chronic damages, such as alcohol, LPS, BDL, drug, CCl_4_ and high fat, leading to the liver injury and fibrosis characterized by the activation of HSCs and the hepatocytes injury, apoptosis or death. Particularly, it could disrupt the circadian rhythm in livers through causing misalignment of the amplitude and phase of a normal rhythm in key cell types, such as HSCs and hepatocytes.

**Figure 3:**
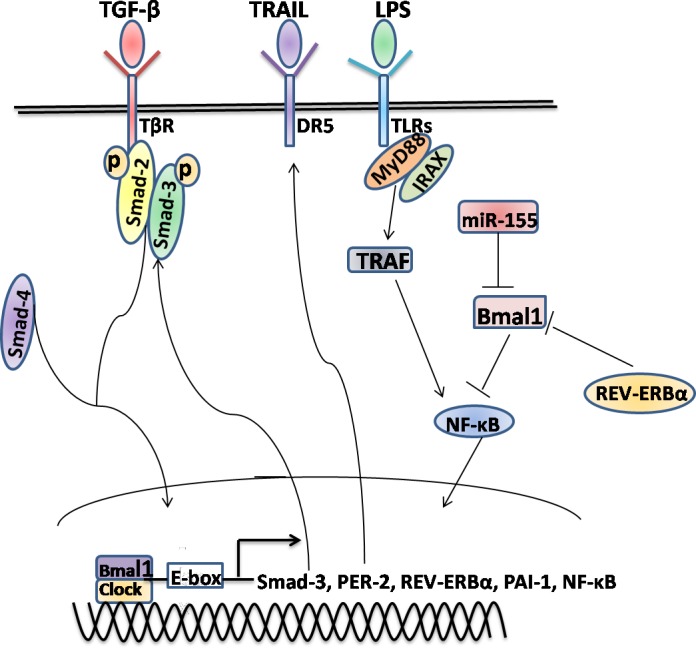
The molecular mechanisms involved in the manipulation of the circadian rhythms in hepatic stellate cell Several of the key signaling pathways, for example TGF-β/Smad, TRAIL/DRs and LPS/TLRs, might either directly or indirectly interact with the circadian rhythms in HSCs *via* regulating the transcription of core clock genes and other CCGs. In addition, the epigenetic modifications, such as miR-155, might be also involved in the rhythmic genes expression. Some other conditions have not yet been described need further study to investigate.

REV-ERBα, a heme-sensing NRs, is involved in metabolic and circadian pathways [119], further suggesting that its up-regulation is a conserved response in activated HSCs and injured livers independent of etiology [120]. Cytoplasmic expression of REV-ERBα was an intrinsic profibrogenic response characterized by the increased fibrogenic markers in activated HSCs [120]. However, it may also have potential antifibrotic functions by repressing the expression and circadian output plasminogen activator inhibitor-1 (PAI-1) through binding the promoter of PAI-1 [121], a coagulation regulator that promotes fibrosis [122]. In line, CLOCK is involved in PAI-1 gene expression and PER2 represses PAI-1 gene transcription in a BMAL1/2-dependent manner [123, 124]. Hence, REV-ERBα is a bifunctional regulator for exhibiting either anti-or profibrogenic effects, depending on milieu. Additionally, REV-ERBα is believed to act as the transcriptional repressor of BMAL1 [125], and the induction of innate immune challenge of macrophages by LPS is conditionally altered by the miR-155/BMAL1/NF-κB axis [126]. LPS and some Toll-like receptors (TLRs) in various cell types, such as HSCs and macrophages, involving in the regulation of liver inflammation and fibrosis [127-129], future researches will continue to focus on expanding our understanding of whether circadian-controlled mechanisms lie it (Figures 2, 3).

## CIRCADIAN RHYTHMS IN LIVER CANCER

Hepatocellular carcinoma (HCC) is one of the most common causes of cancer-related death in worldwide, which molecular pathogenesis is extremely complex and heterogeneous [130, 131]. Evolving information suggests that the incidence and mortality of HCC are expected to rise since the increasingly metabolic syndrome with NAFLD, in addition to viral hepatitis and alcohol-induced liver diseases [130]. In view of the link between disrupted circadian rhythms and NAFLD has been extensively investigated [132, 133], it is of interest to explore the functions and mechanisms of circadian clock involved in HCC.

Several lines of evidence have established functional correlation between the disruption of circadian rhythms and carcinogenesis [134]. For instance, oscillatory genes have the ability to influence the different etiology of cancers, including cell proliferation [135], apoptosis [136], cell cycle control [137], DNA damage response [138] and treatment sensitivity of chemotherapy agents and radiation in cancers [139, 140]. In these backgrounds of divergent cancer types [141-143], observations from cell/animal models and clinical patients have revealed that the expression profiles of circadian genes are quite different between the cancerous and noncancerous samples. In particular, many avenues of research have provided the tantalizing suggestion that hepatocarcinogenesis might directly or indirectly undergo a series of interactions with the circadian clock that can account for oscillator function. A number of mechanisms may explain the circadian control on HCC, firstly, a down-regulated gene, BMAL2 in HCC may exert the effect of inhibiting the cell proliferation, in turn, overexpression of antisense BMAL2 results in reduced cell cycle time and TNF-α-induced increment of CPP32/caspase-3 activity, and a concomitantly increased G2/S phase transition of cells [144]. An increased risk for the development of HCC is closely associated with chronic hepatitis B virus (HBV) infection [145]. Recently, it has identified that the mRNA expression levels of the PER1, 2 and 3 and CRY2 genes in HCC tissue are significantly decreased [146], which might be disrupted by the HBV X protein (HBx). Moreover, the circadian protein PER2 could counteract HCV replication [147], it is therefore supported the hypothesis that mutual effect between viral infection and clock gene machinery which might be implicated in the HCC. Furthermore, mutation of CRY in p53-null mice delays the onset of cancer by sensitizing TNF-α-initiated apoptosis through interfacing with the GSK3β kinase and alleviating prosurvival NF-κB signaling [148]. In consistent, early study observed the decreased levels of both RNA and protein of the BMAL1, CLOCK, PER1, 2, 3, Cry2, CKIε and TIM genes in HCC cells in comparison to their noncancerous counterpart cells [149]. Down-regulation of these circadian genes is likely to be caused by several non-genetic factors, including promoter methylation, overexpression of EZH2 or other factors, which is tightly associated with the disturbance of cell cycle, tumor size and tumor grade [149]. These results in agreement with previous studies that epigenetic silencing modifications, such as DNA hyper-methylation and histone H3 acetylation, are responsible for restraining clock genes expression in the tumorigenesis [150]. Thus, differentially methylated or acetylated genes in HCC as compared to normal samples whether contributing to the circadian clock system need further study to confirm it. Coincidentally, a long noncoding RNA (lncRNA), is highly upregulated in liver cancer (HULC) and believed to contribute to the acceleration of hepatocarcinogenesis through disturbing circadian rhythm of CLOCK and its downstream circadian oscillators, such as PER1 and CRY1 [151]. In other words, down-regulation of several clock genes results in disturbance of circadian rhythm in HCC, which may disrupt the control of the central pacemaker and benefit selective survival of cancerous cells and promote carcinogenesis [152].

Recent data have shown that liver-specific overexpression of ET1 in the zebrafish causes the HCC [153], owing to the disruption of liver clock to some extent [105]. ET1 is significantly up-regulated in HBx-induced HCC in mice [154], whether it influences the circadian rhythm in this process is a major objective in future research. Hepatic metastases of C26 colon carcinoma with a disrupted circadian rhythm phase shift liver tissue clocks, which might contribute to fatigue and sleep disorders in cancer patients [155]. Hypoxia-inducible factor (HIF)-1α and HIF-2α in a hypoxic microenvironment may contribute to the disturbance in the expression of circadian genes in HCC [156]. In addition, genetic variants in circadian genes are significantly associated with susceptibility and prognosis in cancer patients [143]. For example, a single functional polymorphism of PER3 gene (one SNP rs2640908) is significantly associated with increased overall survival in HCC patients treated with transcatheter arterial chemoembolization TACE [157]. This finding from the candidate gene association study provides further confirmation of the role of circadian biomarkers in HCC patients, although warranting further confirmation and mechanistic investigation of other circadian biomarkers in HCC tumorigenesis. Surprisingly, hepatoma is differentially sensitive to circadian timing signals than in healthy tissue in the regard of restricting food availability of daily timed meals [158]. However, on the other hand, controlling mealtiming might be used to increase the efficacy of treatment by many therapeutic cytotoxic drugs [158, 159].

The circadian disruption caused by long-term shift work also increases the risk of the development of HCC. In experimental models of shift-work, circadian clock disruption from chronic jet-lag (CJL) significantly downregulates p53 and upregulated c-Myc [160], thus favoring cellular proliferation in mice exposed to the hepatic carcinogen, diethylnitrosamine (DEN). Based on these results, it might bring to light the clock genes as circadian biomarkers which are indispensable for the molecular mechanism of hepatocarcinogenesis (Table 2).

**Table 2:** Characteristics of various circadian genes involved in hepatocellular carcinoma

Genes	Expression	Function and regulatory mechanisms	References
Bmal1	↓	Influence cell cycle	[149]
Bmal2	↓	Inhibition of cell proliferation and cell cycle; Induction of apoptosis	[144]
Clock	↓	HULC	[151]
Per1	↓	Methylation, HULC	[146, 149, 151]
Per2	↓	Counteract HCV replication; tumor size(>3 cm)	[146, 147, 149]
Per3	↓	Overexpression of EZH2, tumor size(>3 cm)	[146, 149]
Cry1	↓	Induction of cell apoposis, Methylation	[148, 151]
Cry2	↓	Induction of cell apoposis	[146, 148]
CKIε	↓	EZH2	[149]
Tim	↓	tumor grade	[149]

## CONCLUDING REMARKS

Given the importance of internal pacemaker in modulating liver-specific rhythmic programs [161], studies have been increasingly carried out to delineate some new regulatory components of circadian function. As a result, it has provided some novel descriptions of previously unidentified mechanisms by which the circadian clock controls the disparate liver diseases [162]. For example, unlike PER1 and 2, PER3 is not indicated to be necessary for the circadian operation, however, it has been largely accepted that PER3 might correlate with circadian regulation [163, 164]. The robust output of liver circadian clock is derived by the orchestration between central oscillator in SCN and liver-specific local clocks, involving autoregulatory feedback loops and other uncharacterized processes [165, 166]. We highlight the effects of various signaling pathways, TFs and epigenetic mechanisms with the potential to alter the expressions and functions of classic core clock genes and clock-controlled factors at the transcriptional, post-translational and other levels in liver steady-state conditions and diseases. Althoughmolecular mechanisms in relation to circadian rhythms warrant further investigation, they might provide new insights into the development of some chronotherapeutics-dependent approaches to the liver diseases.

### Abbreviations

SCN, suprachiasmatic nucleus; CCGs, clock-controlled genes; CLOCK, circadian locomotor output cycles kaput; BMAL1, brain and muscle Arnt-like protein 1; PER, period; CRY, cryptochromes; NRs, nuclear receptors; ROR, retinoic acid-related orphan receptors; REV-ERB, retinoic acid receptor-related orphan nuclear receptor gene; RORE, ROR response element; TFs, transcription factors; PI3K, phosphatidylinositol 3-kinase; FOXO3, forkhead box class O3; PPAR, peroxisome proliferator-activated receptor; IRS, insulin receptor substrates; GH, growth hormone; TAG, triglyceride; NAFLD, nonalcoholic fatty liver disease; ER, endoplasmic reticulum; SREBP, sterol responsive element binding protein; FGF, fibroblast growth factor; Gys2, glycogen synthase 2; BA, bile acid; HNF4α, hepatocyte nuclear factor 4α; Dbp, D-site binding protein; KLF, krüppel-like factor; MLL, methyltransferase mixed-lineage leukemia; SIRT1, sirtuin 1; miR, microRNA; HDAC, histone deacetylase; ECMs, extracellular matrix; HSCs, hepatic stellate cells; LD, light:dark; ET-1, endothelin-1; LPS, lipopolysaccharide; PMN, polymorphonuclear cells; APAP, acetaminophen; Ucp2, uncoupling protein-2; CDK, cyclin-dependent kinases; PAI-1, plasminogen activator inhibitor-1; HCC, hepatocellular carcinoma; HBV, hepatitis B virus; HBx, HBV X; lncRNA, long noncoding RNA; HULC, highly upregulated in liver cancer; HIF, hypoxia-inducible factor; CJL, chronic jet-lag; DEN, diethylnitrosamine.
